# Microbial biotechnology to assure national security of supplies of essential resources: energy, food and water, medical reagents, waste disposal and a circular economy

**DOI:** 10.1111/1751-7915.14049

**Published:** 2022-03-24

**Authors:** Kenneth Timmis, Juan Luis Ramos, Willy Verstraete

**Affiliations:** ^1^ Institute of Microbiology Technical University Braunschweig Germany; ^2^ Estacion Experimental de Zaidin CSIC Granada Spain; ^3^ Center for Microbial Ecology and Technology (CMET) Ghent University Ghent Belgium

## Abstract

The core responsibility of governments is the security of their citizens, and this means *inter alia* protecting their safety, nutrition and health. Microbiology and microbial biotechnology have key roles to play in improving supply security of essential resources. In this paper, we discuss the urgent need to fully and immediately exploit existing microbial biotechnologies to maximize supply security of energy, food and medical supplies, and of waste management, and to invest in new research specifically targetting supply security of essential resources.

The tragedy of the invasion of the Ukraine has not only heaped death, injury and misery on an innocent and unsuspecting population, ripped gaping holes in families, displaced whole communities and created an enormous number of refugees, and provoked all the stress and mental health problems associated with these issues, but follows closely on the heels of the other, still ongoing, global tragedies of COVID and the global warming‐caused calamities of fires, floods and landslides. What further horrors are in the wings, just waiting for a nudge? More importantly, what can/should we do to prevent, or at least mitigate, the nudge and its consequences? Here, we argue that reducing dependencies on global supply chains is a crucial component of efforts to reduce exposure to catastrophes.

The Ukraine tragedy has exposed full frontal the catastrophic dangers of dependency on global supply chains of essentials, not only because of their lack of security but also because they can severely compromise the political decision‐making process at national and regional levels, and hence geopolitics, and thereby directly impact the formulation of policies needed to counteract threats to world peace.

The immediate example is energy security that is directly impacted by Russian gas and oil supplies. On one hand, many countries depend on this source, which thereby yields enormous revenue that in part pays for the current military activities we observe. On the other, attempts to discourage these activities through sanctions are mired in national conflicts of interest, making the orchestration of a unified response challenging.

Furthermore, a major challenge to food security for many nations is about to unfold as a result of the Ukraine tragedy, since the Ukraine and Russia are major exporters of grain and grain products (https://www.reuters.com/business/russia‐ukraine‐conflict‐highlights‐wheat‐supply‐vulnerability‐2022‐03‐03/; https://www.bloomberg.com/news/articles/2022‐03‐02/russia‐s‐war‐with‐ukraine‐could‐devastate‐global‐grain‐markets ; https://www.investmentmonitor.ai/special‐focus/ukraine‐crisis/countries‐exposed‐ukrainian‐food‐exports; https://www.spglobal.com/commodity‐insights/en/market‐insights/latest‐news/agriculture/022422‐factbox‐russias‐ukraine‐invasion‐seen‐disrupting‐vegetable‐oil‐grain‐trade‐flows).

The core responsibility of governments is the security of their citizens, and this means *inter alia* protecting their safety, nutrition and health. Uncritical embracing of the global economy – buy where cheapest, sell everywhere possible, externalize environmental costs (https://www.unep.org/news‐and‐stories/press‐release/new‐study‐shows‐multi‐trillion‐dollar‐natural‐capital‐risk; https://www.imf.org/external/pubs/ft/fandd/basics/external.htm), dispose of problems where cheapest (e.g. see: http://sdg.iisd.org/news/sdg‐index‐report‐calls‐for‐eu‐wide‐vnr‐by‐2023/) – has created all manner of problems, witness the

- horrendous problem of plastics in all forms dispersed all over the globe;
- practice of getting rid of waste by exporting it – out of sight, out of mind, with little or no regard to its fate (http://sdg.iisd.org/news/sdg‐index‐report‐calls‐for‐eu‐wide‐vnr‐by‐2023/);
- destruction of rain forests;
- local pollution generated by some mining operations;
- almost inhuman entrapment of workers in e‐commerce activities;
- and, perhaps most importantly, the inhuman exploitation of child labour in mining and other commercial operations

And: outsourcing production to far‐away countries and the associated transportation comes with a significant depletion of local knowledge and skills, an ever‐increasing carbon footprint, and the need for unnecessary logistical infrastructure and additional regulatory oversight.

Governments can and must minimize insecurities in, and thus dependencies on others for, *essential* resources by maximizing own production. This is true of supplies of energy, food, medical products and so on. It is not a question of being entirely self‐sufficient/completely independent of external supplies, which is obviously not possible for most essential resources in most countries; it is a question of minimizing the problem by reducing dependencies where possible, by stimulating local creativity and exploiting own potential.

Yes: this will involve an economic cost, at least initially, but dealing with the current energy crisis and looming grain crisis also has an economic cost, a cost that can be repeated at any time in the future. And, as we know, as expertise and manufacturing efficiencies increase, costs come down. Moreover, as we also know, existing prices depend on supply and demand, so also change, sometimes unpredictably, over time.

While diverse players and strategies must be involved in creating the political, economic, technological and logistical framework needed to maximize national security by minimizing dependencies upon others for essential resources, it is crucial to look beyond what may be possible now and strategically invest in the future.

Microbiology and microbial biotechnology have key roles to play in improving supply security of essential resources (Timmis et al, 2017; see also the Special Issue of Microbial Biotechnology on *The contribution of microbial biotechnology to sustainable development goals*: https://sfamjournals.onlinelibrary.wiley.com/toc/17517915/2017/10/5). These roles, some of which can immediately be more effectively exploited than at present, and others that need further development, include but are not limited to applications in the following resource sectors:

## Energy

There is a severe crisis in the supply of fossil fuels in many parts of the world. Important alternatives to fossil fuels are wind/wave, solar, in some countries nuclear energy, and biofuels (see accompanying article by Ramos *et al*., 2022). Bioelectricity may also become a practical option in the future. Wave energy obviously only works for countries with significant lengths of coastline, and solar only works well at lower latitudes, so individual circumstances dictate to some extent the options available. However, all countries produce waste and have possibilities for energy crops, and can thus produce methane gas – an important fuel – by fermentation (Verstraete *et al*., 2022). Countries at higher latitudes may not have good conditions for production of solar energy, but usually have good conditions for growth of softwoods, etc., which can be used to produce biofuels by microbiological processes.

A number of programmes for biofuel production from biomass and municipal solid wastes have been implemented in several countries (mainly Brazil, the United States and European Union) with the aims of not only of enhancing fuel supplies and of reducing the importation of fossil fuels, but also of reducing greenhouse gas emissions. Some biofuels like butanol can be distributed through existing petroleum systems. Hence, technology and logistic infrastructures are available for a secure worldwide biofuel production.

Current microbial processes for the production of biofuels and electricity may not always be economically competitive with fossil fuels under recent *normal* circumstances, but we see right now that abnormal circumstances can dramatically change economics (and politics). In addition, economics change with technology development and scale. What is desperately needed is the immediate scale‐up of existing technology that works well – biogas, bioalcohols and biodiesel technologies are prime examples – and massive investment in research to advance other promising technologies, so that our present dependencies progressively decrease.

## Food

The economics of the food value chain is such that global supply chains determine food sources. For example, in many countries, imported milk is much less expensive than home‐produced milk, so to some extent the whole milk value chain is outsourced. While *facultative* food items – Roquefort cheese, Jamon Iberico, Parma ham and so forth – are by definition items that cannot be produced in other countries, they do not represent an issue of food security, which is concerned with *essentials*, like grains, vegetables, fruit and meat.

Governments ignore the domestic potential to grow essential food crops at their peril. Again: the economics of food sources abroad may appear attractive, though only in times of beneficial climate and geopolitical calm. However, we are in the middle of a global warming development whose future progression is uncertain but its associated climate change is already having major negative impacts on farming by promoting drought, extreme weather events, unseasonal temperatures that can *inter alia* result in higher levels of crop pests that reduce harvest yields, and accelerate degradation of farmland already made vulnerable through over‐exploitation (Timmis and Ramos, 2021). This, and political instability, can radically change both the economics and security of food supply chains. When this happens, re‐configuring domestic land for the production of vital crops and animal‐related products takes considerable time during which insecurity and hardship prevail.

It is therefore essential for governments to maximally exploit with urgency their own natural resources – land and water resources (the *water footprint* issue is now well established, it must receive much more attention, and be better managed – see, e.g. Verstraete *et al*., 2022), the plants it can grow, and the food animals it can support – and thereby minimize food insecurity in the long term. For countries with soils containing legacy pollutants or that are degraded, this also means soil remediation, regeneration, restoration and protection, in order to increase the surface area of agricultural land that can be used for food production (Timmis *et al*., 1994; Lal, 2004; Maestre *et al*., 2017; Bonfante, *et al*., 2020; Bardgett and Van Wensem, 2021; Timmis and Ramos, 2021; Verstraete *et al*., 2022), without destroying forests that harbour fixed carbon and biodiversity. And all countries, including and especially those lacking adequate land and water resources suitable for farming, should where possible embrace vertical farming and aquaponics to increase food production potential.

Meat production is important because it is the major source of essential dietary protein for most of us, but of course comes with a high carbon footprint. Current microbiome research suggests that it may be possible to increase meat yields and reduce greenhouse gas emissions per animal, so investment in this may result in significant carbon footprint reductions.

However, there are other, lower carbon footprint sources of dietary protein: *microbes*. Microbial protein in the form of edible mushrooms has been consumed for millennia, and fungal meat substitute since the 1980s, but other microbial dietary protein options are now being vigorously explored, in part galvanized by the combined spectres of exhaustion of natural resources by the growing world population, and climate change resulting in part from greenhouse gas emissions from ruminants.

There is a growing number of options for the production of microbial dietary protein (Choi *et al*., 2022). Autotrophic microbes are of course at the base of many food webs, so their serving directly as food for us is a very natural solution. One option is a particularly beautiful route: the use of green energy to electrolyse water, and subsequent use of the hydrogen and oxygen, plus CO_2_, to grow hydrogenotrophic microbial biomass (Verstraete *et al*., 2022). The route is known and the economics are feasible, especially if the environmental costs of the conventional production of proteins are taken into account and not externalized (https://www.unep.org/news‐and‐stories/press‐release/new‐study‐shows‐multi‐trillion‐dollar‐natural‐capital‐risk; https://www.imf.org/external/pubs/ft/fandd/basics/external.htm), as is now common practice.

In all these aspects – farming itself, land restoration and protection, vertical farming, and so forth – microbes not only play a key role in the health of the plants and animals, and the cultivation systems, but also can be exploited to improve performance, i.e. increase food yields, without increasing the use of polluting agrochemicals. In addition to promoting plant growth by providing fixed nitrogen and accessing phosphorus, microbes can play a major role in plant and food animal protection from pests and disease (Mendez *et al*., 2011; Pérez‐García *et al*., 2011; Ruffner *et al*., 2012). And of course they are the food itself in the case of microbial protein. Microbial technologies are thus pivotal to maximization of food yields.

## Medical supplies

The covid pandemic exposed major bottlenecks in global medical supplies. In terms of microbiology, vaccines, many therapeutic drugs, and diagnostic reagents are based on microbial substances produced by fermentation. The pharmaceutical industry is truly global with different components needed for the manufacture of individual drugs and vaccines often being produced in different countries, and for any product, the weakest link in the chain determines the supply security. Generics, for example, are mostly produced in countries where labour is least expensive. Governments should where possible organize locally‐produced vital components that provide a higher degree of security for essential goods. This may involve incentives that encourage the creation of new local industries, or partnerships with existing enterprises to manufacture components that are otherwise economically uncompetitive on the world stage. In terms of vaccines, drugs and diagnostics, this may involve investment in fermentation capacity. In any case, it requires significantly more investment in research and innovation.

## Waste and recycling

Waste has traditionally been perceived as something to be disposed of, and much of it is channelled into the global supply‐disposal chain – with unforeseen consequences. However, microbes are able to transform many organic wastes and these are now perceived as organically rich resources that can be used as feedstocks for upscaling to valuable products (Verstraete *et al*., 2022). It is crucial that governments prohibit the export of wastes that create environmental problems elsewhere, disallow externalization of environmental costs in accounting exercises and financial audits (and thereby create objectivity and transparency in cost comparisons of new and existing technologies), and vigorously pursue the development of microbially‐based processes that transform them into useful products.

In addition, the current attitude towards non‐biodegradable consumer products, particularly towards conventional plastics, must change dramatically. There was a time when our planet was threatened by fluorinated hydrocarbons that degraded the protective ozone layer. Fortunately, it was possible to prohibit them worldwide. In a similar way, time has come to address all consumer products which have a risk of being dispersed in the environment. They should from now on be biodegradable by microbes, for instance at a properly justified minimal rate, e.g. over a period of several years, and managed domestically. The time has come to take a bold action on this matter: recycling *sensu largo*, either by engineered or by natural processes, should be feasible and fully imposed on all products entering the environment.

Finally, it is essential to bring greater focus on our environment, the vital services it provides, its degradation and that of its services by legacy and current pollution, and ways and means of protecting and repairing it. One key action will be to create international research programmes and centres of excellence that serve as beacons for outstanding research that creates new and innovative solutions to environmental problems, and go‐to places for policy advice and know‐how (see accompanying article by Timmis and Verstraete, 2022).

## Conclusions and recommendations

Obviously, the overwhelming importance of increasing the security of essential supplies concerns many things, but the principles are the same. For most essential resources and most countries, this is not about self‐sufficiency but rather about maximally reducing exposure to insecurities, in order to minimize shortages and disruptions when they occur.

We have focussed here on four key areas: energy, food and water, medical supplies and waste disposal, because microbiology plays a pivotal role in all of them, but microbiology also plays a role in the provision of other essential resources like clean water, chemicals and materials.

What is absolutely essential and urgent is:

1. ***An audit of resource production potential***. Each country should carry out a transparent, unbiased (no sacred cows) and comprehensive audit of its potential to domestically produce energy, food and strategically important medical components, create a national production plan that maximally exploits this potential, and promptly implement the plan so that knowledge and skills are present, diversified and assured for future generations.
2. ***An audit of waste disposal and circular economy potential***. Each country should examine its potential to recycle its resources (water, nutrients and wastes) and prohibit the export of wastes. Moreover, externalization of costs to the environment and to future generations must end. Each country should carry out a transparent, unbiased (no sacred cows) and comprehensive audit of its potential to upscale its wastes and safely dispose of those that cannot be upscaled, create a national waste disposal plan that maximally exploits this potential and promptly implement the plan. The problem of dispersal of plastics – macro and micro – in the environment must be addressed with great determination to finally resolve it adequately.
3. ***Substantive investment in ‘essential resource security research’***. It is paramount that governments recognize that the future is not readily predicted by the present and that advances in research can radically and rapidly change technological, economic and political realities. Thus, for example, the current competitive disadvantage of biofuels can disappear with technological advances, political crises of the type we are currently experiencing, and changes in other fields not obviously linked to energy at the moment. Thus, it is crucial that governments identify key priorities, such as energy security, and then invest substantially in research directed at making promising approaches better, more efficient and more economical. Topics we have identified here that are microbiology‐centric are
  - biofuels,
  - agriculture: plant–microbe interactions that determine yield, biological nitrogen fixation, plant–microbe–soil interactions that improve soil quality and restore degraded soils, vertical farming and aquaponics, higher yielding, less polluting meat production,
  - dietary protein: microbial protein based on green energy,
  - medical components, such as vaccines, drugs and diagnostic reagents
  - waste management starting from the design of the commodities and implementing full recycling as part of the overall value chain
4. ***Creation of supportive environments (educational, financial and regulatory) for innovation: an audit of innovation barriers***. First and foremost, we must educate the citizen about all these matters, preferably already in school (e.g. see Timmis *et al*., 2019): informed public support is essential for our democratic governmental platforms. In addition, as we have seen with the digital revolution, electric cars and so on, governments do not play a major role in reduction to practice and commercial implementation of new discoveries; exceptional entrepreneurs do. However, entrepreneurs often face severe hurdles in the innovation pathway because they are competing against established technologies and economic and regulatory frameworks established around existing technologies. In order to facilitate the transfer of new technologies to market and their scale‐up to reduce costs and improve their market competitiveness, it is essential to lower existing barriers to market penetration, while of course maintaining public safety.

Technology can advance stochastically, so it is often difficult to predict where the most important new advances will occur next and to specifically tweak the barriers related to these. Therefore, barriers in general should be minimized. To do this, a comprehensive audit of innovation barriers conducted hand in‐hand with stakeholder entrepreneurs, should be made, proposals to reduce those that are not absolutely essential should be formulated and the proposals promptly implemented. Regulatory frameworks are essential but, more than ever before, they need to be dynamic and actively stimulate important new developments for the better of everyone.

## Conflict of Interest

None declared.
